# SRRM4 Expands the Repertoire of Circular RNAs by Regulating Microexon Inclusion

**DOI:** 10.3390/cells9112488

**Published:** 2020-11-16

**Authors:** Vanessa M. Conn, Marta Gabryelska, Shashikanth Marri, Brett W. Stringer, Rebecca J. Ormsby, Timothy Penn, Santosh Poonnoose, Ganessan Kichenadasse, Simon J. Conn

**Affiliations:** 1 Flinders Cancer Research, College of Medicine and Public Health, Flinders University, Bedford Park 5042, South Australia, Australia; vanessa.conn@flinders.edu.au (V.M.C.); marta.gabryelska@flinders.edu.au (M.G.); shashikanth.marri@flinders.edu.au (S.M.); brett.stringer@flinders.edu.au (B.W.S.); rebecca.ormsby@flinders.edu.au (R.J.O.); timothy.penn@flinders.edu.au (T.P.); 2 Flinders Health and Medical Research Institute (FHMRI), College of Medicine and Public Health, Flinders University, Bedford Park 5042, South Australia, Australia; santosh.poonnoose@flinders.edu.au (S.P.); ganessan.kichenadasse@flinders.edu.au (G.K.); 3 School of Clinical Medicine-Greenslopes Clinical Unit, The University of Queensland, Brisbane 4120, Queensland, Australia; 4 Department of Neurosurgery, Flinders Medical Centre, Bedford Park 5042, South Australia, Australia; 5 Department of Medical Oncology, Flinders Centre for Innovation in Cancer, Flinders Medical Centre, Bedford Park 5042, South Australia, Australia

**Keywords:** circular RNAs, alternative splicing, splicing factors, microexons, SRRM4, glioblastoma

## Abstract

High-throughput RNA sequencing (RNA-seq) and dedicated bioinformatics pipelines have synergized to identify an expansive repertoire of unique circular RNAs (circRNAs), exceeding 100,000 variants. While the vast majority of these circRNAs comprise canonical exonic and intronic sequences, microexons (MEs)—which occur in 30% of functional mRNA transcripts—have been entirely overlooked. CircRNAs which contain these known MEs (ME-circRNAs) could be identified with commonly utilized circRNA prediction pipelines, CIRCexplorer2 and CIRI2, but were not previously recognized as ME-circRNAs. In addition, when employing a bespoke bioinformatics pipeline for identifying RNA chimeras, called Hyb, we could also identify over 2000 ME-circRNAs which contain novel MEs at their backsplice junctions, that are uncalled by either CIRCexplorer2 or CIRI2. Analysis of circRNA-seq datasets from gliomas of varying clinical grades compared with matched control tissue has shown circRNAs have potential as prognostic markers for stratifying tumor from healthy tissue. Furthermore, the abundance of microexon-containing circRNAs (ME-circRNAs) between tumor and normal tissues is correlated with the expression of a splicing associated factor, Serine/arginine repetitive matrix 4 (*SRRM4*). Overexpressing SRRM4, known for regulating ME inclusion in mRNAs critical for neural differentiation, in human HEK293 cells resulted in the biogenesis of over 2000 novel ME-circRNAs, including *ME-circEIF4G3*, and changes in the abundance of many canonical circRNAs, including *circSETDB2* and *circLRBA*. This shows SRRM4, in which its expression is correlated with poor prognosis in gliomas, acts as a bona fide circRNA biogenesis factor. Given the known roles of MEs and circRNAs in oncogenesis, the identification of these previously unrecognized ME-circRNAs further increases the complexity and functional purview of this non-coding RNA family.

## 1. Introduction

In its simplest terms, RNA splicing converts precursor mRNA into mature mRNA through the removal of introns and sequential ligation of canonical exons. The RNA splicing machinery, called the spliceosome, is a large and dynamic ribonucleoprotein complex consisting of small nuclear RNAs and approximately 80 distinct proteins [1,2]. Like any enzymatic process, RNA splicing is affected by the accessibility and structure of its substrate, in this case, the 5′ and 3′ ends of an intron by scanning for the canonical GU and AG splice sites, respectively. However, non-canonical RNA transcripts are frequently made through a process called alternative splicing as a result of many factors including RNA secondary structure, strength of splice recognition sites and dynamic permutations of RNA and protein factors within the spliceosome [3,4]. It is estimated that 90–95% of all eukaryotic genes are alternatively spliced, and this represents the greatest source of expansion of the functional transcriptome and proteome.

Alternative splicing is a dynamic, well-orchestrated, and cell-type specific process which play critical roles in cell differentiation, maintenance of stem cell pluripotency [5] and generation of highly tissue-specific proteins, particularly in the brain which has the highest rate of alternative splicing in the human body [6]. Perhaps unsurprisingly, misregulation of alternative splicing through mutations of RNA splicing factors and RNA splice sites can be devastating, with evidence of aberrant splicing found in cancer and in neurological diseases [7].

The most common alternative splicing events in humans are exon skipping/inclusion (>40% of variants), use of alternative splice sites, and intron retention. While the size of exons and their flanking introns can impact the profile of alternatively spliced transcripts, in the central nervous system, it has been reported that the inclusion of short exons is more frequent [8,9]. The most highly conserved component of neuronal alternative splicing across development are extremely short exons, known as microexons (MEs). Herein, we utilize the commonly employed definition of MEs as those exons 3–30 nucleotides (nt) in length [3,10].

MEs were originally found in 1985 as two 5 nt exons in the Drosophila homeotic *Ubx* gene. While found sporadically since that time, it was not until 2003 through a combination of bespoke computational analysis on a reference databases of full-length mRNA sequences, that MEs were systematically identified in a range of model species, including humans [11]. At this time, 223 MEs were identified (~1.6% of human genes), yet more contemporary estimates with the aid of high-throughput RNA sequencing have expanded these numbers dramatically to 2008 (in 1587 genes) [12] and over 13,000 for exon sizes up to 51 nt [13].

While still underappreciated, a focus of research into MEs has been in neural development and disease [14]. Although there exists a number of neural-specific RNA splicing factors which affect alternative splicing, including RBFOX1, SRSF11, and PTBP1/2, most neural MEs are regulated by the Serine/arginine repetitive matrix 4 or Neural-specific serine/arginine repetitive splicing factor of 100 kDa (SRRM4/nSR100), through its binding to adjacent intronic enhancer motifs [15,16]. SRRM4 is specifically expressed in the brain and sensory organs and is highly conserved across vertebrates, but lacking in invertebrates, perhaps associated with the increased functional complexity of the vertebrate nervous system [17].

Reinforcing SRRM4 as a master regulator of alternative splicing in the brain, it has been shown to regulate conserved networks of alternative splicing events in human and mouse neural functions including cytoskeletal organization and GTPase signaling [14]. One critical event mediated by SRRM4 is the increased expression of another neural-specific splicing factor, Ptbp2, by regulating the inclusion of exon 10 to prevent nonsense mediated decay driven turnover of *Ptbp2* mRNA [17]. However, the most widely known manifestation of SRRM4-regulated alternative splicing is the inclusion of exon 4 in the mRNA of the REST/NSRF transcriptional repressor [18]. This REST/NSRF variant, called *REST4*, encodes an inactive protein lacking five critical zinc finger DNA binding domains [19,20]. This relieves the transcriptional silencing of a subset of transcripts, allowing neuronal maturation and differentiation to proceed. Therefore, alternative splicing and MEs mediated by SRRM4 are critical in the brain.

Like MEs, circular RNAs (circRNAs) were originally largely dismissed as outliers of alternative splicing from their initial visual identification in the 1970s [21], and their sequence-based identification in the 1990s [22]. However, with the synthesis of high-throughput RNA sequencing and custom bioinformatics pipelines, circRNAs have been identified as the most contemporary class of alternatively spliced, largely non-coding RNAs, with current estimates of over 100,000 unique circRNAs within the human transcriptome [23,24].

**Figure 1:**
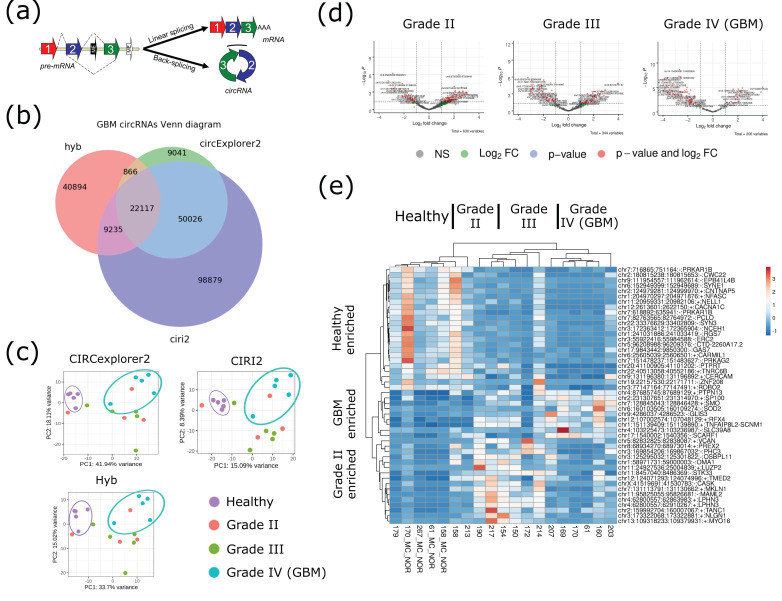
Circular RNA (circRNA) profiling of human gliomas can stratify glioblastoma (GBM) tumors. (**a**) Canonical linear splicing versus non-canonical, back-splicing producing mRNA and circRNA, respectively. Arc highlights the location of the backsplice junction. (**b**) Scaled Venn diagram showing total number of circRNAs identified from all 19 brain, circRNA-seq libraries using three different circRNA identification pipelines, CIRCexplorer2, CIRI, and Hyb. (**c**) Two-dimensional PCA plot utilizing circRNA-seq output from CIRCexplorer2 on 19 samples across healthy, grade II, III, and IV (GBM) gliomas. Ellipses included to highlight stratification of healthy tissue (purple) and grade IV tumor (blue) for each of the three circRNA prediction pipelines. (**d**) Expanded volcano plot showing circRNAs regulated between healthy tissue and grade II (left panel), grade III (middle panel) and grade IV, GBM (right panel). (**e**) Heat map showing representative examples of circRNAs specific to healthy brain tissue, grade-specific (grade II and grade IV (GBM)) using CIRCexplorer2. The color scale bar represents the log-transformed normalized reads.

CircRNAs are formed co-transcriptionally and their biogenesis is in competition with canonical linear RNA splicing (Figure 1a) [25]. CircRNA biogenesis is regulated primarily by two non-mutually exclusive mechanisms: (1) the presence of inverted, complementary repeats in flanking introns; and (2) interaction with RNA binding proteins to promote (QKI, FUS, MBNL) or inhibit (ADAR1, mediating adenine to inosine editing) production [23,24,26,27]. In addition, the rate of RNA polymerase II elongation and flanking intron size has also been shown to be positively correlated with circRNA formation from distinct loci [28,29].

It has emerged that alternative splicing is a critical factor in the initiation and progression of human pathologies, including cancer [30,31]. Mutation, or mis-regulation, of splicing factors and snRNAs [32] have been found in various cancers, including the most lethal brain cancer, glioblastoma (GBM). Patients diagnosed with GBM have, on average, 15 months post-diagnosis survival and a 5-year survival rate below 10%. Standard therapy involves maximal, yet incomplete, surgical resection in combination with chemotherapy (Temozolomide) and/or radiotherapy. However, despite advances in all of these areas, the survival from GBM has increased only 1% in the past 30 years, compared with 20% for all other cancers [33]. Alternative splicing signatures have been proposed as a novel prognostic marker, but also targeting alternative splicing in GBM could represent a novel and desperately needed therapeutic strategy [34,35]. CircRNAs have been systematically identified in GBM, with a number of examples of circRNA candidates contributing to hallmarks of the disease [36]. However, further investigation into the fundamental basis of alternative splicing in GBM and profiling circRNAs at various stages of progression of GBM is required.

A number of protein factors have been identified that contribute to circRNA biogenesis and a variety of circRNAs have been identified containing permutations of canonical exons [37]; yet there has been little evidence for the inclusion of unique exons in circRNAs as a result of these protein splicing factors. While upregulation of SRRM4 has been reported to promote neuroendocrine prostate cancer through alternative splicing [38,39], SRRM4 is seen to decrease in expression within the tumor proper compared with the leading edge and infiltrating tumor in GBM based on RNA sequencing data from the IVY Glioblastoma Atlas Project [40]. However, its potential role in brain cancer still remains largely unexplored. Here, to our knowledge, we provide the first report of MEs in circRNAs and extend this by identifying SRRM4 as a master regulator of ME inclusion in GBM and as a bona fide circRNA biogenesis factor.

## 2. Materials and Methods

### 2.1. Human Ethics Approval and Patient Tissue Samples

For all primary tissue sourced for these experiments, written informed consent was obtained from each subject or from their guardian. Specimens were received from the SA Neurological Tumor Bank (SANTB) which is supported by Flinders University, Flinders Foundation and The Neurosurgical Research Foundation. The ethics for this project is approved by the Central Adelaide Local Health Network Human Research Ethics Committee (CALHN HREC) with approval number HREC/17/RAH/284. The SANTB is approved by the Southern Adelaide Clinical Human Research Ethics Committee (SAC HREC) with approval number 286.10. Resected tumor tissue was processed in theatre, placed into sterile cryovials and immediately snap-frozen in liquid nitrogen to preserve RNA integrity and histology. Any non-pathological (normal) brain tissue that was removed during surgery, in order to access the tumor, was collected and processed in a similar manner. Clinical grading was performed by SA Pathology and classified according to the WHO classification of tumors of the central nervous system [41].

### 2.2. Circular RNA (circRNA) Sequencing

CircRNA sequencing was performed on 20 tissue samples collected from the South Australian Neurological Tumor Bank with informed patient consent. This included 15 primary gliomas, with five grade II, five grade III, and five grade IV (GBM) tumors as determined by histopathological grading [41] and 5 healthy brain tissue samples (hereafter referred to as healthy, or control tissue; Table 1).

RNA was harvested 48 h post-transfection using TRIzol™ (ThermoFisher Scientific, USA) & Direct-zol™ RNA miniprep kit (Zymo Research, USA) with on-column DNase I treatment. Ten micrograms of RNA were digested with 20 U Ribonuclease R (Lucigen, USA) at 37 °C for 30 min. RNA was subsequently purified by RNA MinElute columns (Qiagen, USA) as per Conn et al. [26]. RNA-seq libraries were prepared using Illumina Truseq Stranded Library Preparation Kit with Ribozero Gold rRNA depletion (Illumina, USA). Raw and processed data was deposited in GEO database for HEK293 SRRM4 overexpression data (accession number GSE148813) and glioma circRNA-seq data (accession number GSE159260).

### 2.3. CircRNA Prediction

Reads were mapped against the human reference genome (hg19) using the STAR spliced alignment algorithm [42] (version 2.5.3a with default parameters and --chimSegmentMin 20) returning an average unique alignment rate of 79% and 81% for total RNA-seq and circRNA-seq, respectively (Table 1). The resulting STAR produced Chimeric.out.junction file for each sample was parsed and annotated for circRNA prediction and backsplice abundance using CIRCexplorer2 [37]. CIRI2 was used to identify the back-spliced junction reads with default parameters [43]. Bedtools were used to find overlapping microexons in circRNAs based on the genomic coordinate locations [44]. In parallel, reads were processed through the Hyb pipeline [45] utilizing the reference Exon-Intron database from Shepelev et al. [46] and adapted to fit the Hyb-preferred database format by incorporating fragment length and retaining strand information. This database was prepared for Hyb as per Travis et al. [45] The Hyb output format included information about exon number, length, start/end positions in the read and start/end positions in the reference sequence. Junction sites representing circRNAs were established as reads where the end of a downstream exon is followed by beginning of the same or upstream exon.

### 2.4. ME-CircRNA Prediction

For identifying ME-circRNAs, we utilized the known MEs present in linear RNAs from Irimia et al. [15]. Firstly, we assessed if these known MEs were expressed in our data by assessing if reads from the fastq file could be found crossing the ME and at least one flanking exon of the same gene. This was then identified as a ME-circRNA if this ME overlapped a circRNA called by the same pipeline on the same strand. If the ME comprised either the 5′ or 3′ exon of the circRNA, it was called as the backsplice junction, whereas, if it was within the circRNA coordinates but not at either termini, it was referred to as being in the body of the circRNA. This was performed for CIRCexplorer2, CIRI2, and Hyb pipelines.

To identify novel ME-circRNAs, at the beginning of the Hyb analysis we permitted reads of 3-30 nt in-between all chimeric reads. Once identified, the chimeric reads identified as backspliced circRNAs were extracted and filtered to determine microexon length (if present) and sequence for further mapping and analysis of these ME-circRNAs. Calculations of circRNA counts was performed using the merging program within the Hyb package. Format of Hyb-identified circRNAs was then transformed into the more widely used output format of CIRCexplorer2 to contain information on chromosome location, gene name, count, and included exons. This format was used to compare the results to CircExplorer2 and CIRI2. The Hyb package is available at Github (https://github.com/gkudla/hyb), and codes can be provided upon reasonable request.

### 2.5. Differential Expression Analysis

Prior to differential expression analysis, circRNAs with very low counts across all the libraries were removed. Low count filtering was done using CPM (Counts Per Million) values rather than counts in order to avoid preference to samples with larger library sizes. Using the edgeR package [47], we calculated median of library sizes and kept genes that have CPM of ≥0.01 in at least three samples for statistical evidence without loss of information [48].

The filtered read counts were normalized using DESeq2 median of ratios [49] method and relative log transformation [50] utilized to generate the PCA plots. For the differential expression, DESeq2 [51] statistical package was used to assess differential gene expression of circRNAs with Log2fold > 1 and *p*-adjusted < 0.01. Volcano plots were generated with EnhancedVolcano R package [52] and heatmaps generated using normalized counts in R. For the circRNAs found in common between all methods, the log-transformed normalized values (CPM) were extracted and plotted on the heatmap using Pearson correlation distance and average linkage method for clustering columns and rows [53].

### 2.6. Cell Culture

HEK293 cells were cultivated at 37 °C with 5% CO_2_ in air in DMEM (Sigma-Aldrich, USA) supplemented with 10% *v/v* FBS (Bovogen, Australia) and 1 mg/mL Antibiotic-Antimycotic (Sigma-Aldrich, USA). Cells were passaged with TrypLE express (ThermoFisher Scientific, USA).

### 2.7. Cloning Overexpression Constructs

Full-length SRRM3 (HsCD00813375) and SRRM4 (HsCD00295084) in pENTR223.1 were obtained from DNASU Plasmid Repository. The coding sequence of each gene, lacking the termination codon, was amplified from this vector using Phusion DNA polymerase (New England Biolabs, USA). The PCR products and pcDNA3.1::FLAG [26] were digested with *Eco*RI and *Eco*RV (New England Biolabs, USA), ligated, and transformed into TOP10 chemically competent *E. coli*. Sanger sequencing was performed to validate the overexpression construct. Overexpression of *ME-circEIF4G3* was performed by using 800nt, reverse complementary introns flanking the circRNA exons (Exons 6a-10) and cloned into pcDNA3.1::FLAG as per [26,54]. Sanger sequencing was performed to validate the overexpression construct. HEK293 cells were transiently transfected with expression constructs using Lipofectamine 2000 (ThermoFisher Scientific, USA) in 6-well plates (Sarstedt, Australia).

### 2.8. RT-PCR/qRT-PCR

RNA, either total RNA or RNase R-treated RNA, harvested using TRIzol™ (ThermoFisher Scientific, USA) & Direct-zol™ RNA miniprep kit (Zymo Research, USA) with on-column DNase I treatment was reverse transcribed with QuantiTect^®^ reverse transcription kit (Qiagen). After RT, QuantiTect SYBR^®^ Green PCR Kit (Qiagen, USA) was used for qRT-PCR as per [55]. RNA was quantified by NanoDrop One Microvolume UV-Vis Spectrophotometer (ThermoFisher Scientific, USA). All oligonucleotides used in this study were ordered from Integrated DNA Technologies (Singapore), with sequences provided (Table S1).

### 2.9. Western Blotting

Total soluble protein was harvested from HEK293 cells using RIPA buffer with 1× protease inhibitor cocktails (mini-EDTA free) and phosphatase inhibitor cocktails (ThermoFisher Scientific, USA) with western blotting performed as per Conn et al. [26]. Anti-FLAG M2 antibody (F1804; Sigma-Aldrich USA) was used at 1:2,500 dilution; with goat anti-mouse HRP conjugated secondary antibody (ThermoFisher Scientific, USA) at 1:10,000 dilution. Chemiluminescent detection was carried out using Super Signal West Pico PLUS (ThermoFisher Scientific, USA) and Precision Plus Protein™ Kaleidoscope™ Prestained Protein Standard (Bio-Rad, USA) was used for size estimation.

## 3. Results

### 3.1. Circular RNA Sequencing across Human Glioma Grades and Control Brain Tissue

Purified RNA from healthy and glioma tissues underwent digestion with Ribonuclease R (RNase R) to degrade linear RNAs and enrich for circRNAs. Validation of this digestion was achieved by performing RT-PCR on cDNA from RNase R-digested, or mock-treated RNA. In each case, the linear RNA targets were depleted following RNase R digestion, while known circRNA targets could be amplified (Figure S1a). The RNA was depleted for ribosomal RNA and RNA-seq libraries were prepared with the Truseq Stranded Total RNA kit and sequenced by NextSeq 550 using the High output 300 cycle kit, 150PE (Illumina, USA). CircRNA-seq libraries had an average read depth of 27 million reads, with 85% mapped reads to human genome assemble hg19. One library (Sample #267, grade II glioma) was excluded due to low percentage of mapped reads (39.1%) and significantly distinct circRNA profile from principal component analysis (PCA) on normalized circRNA counts (Figure S1c). Therefore, all further analyses were performed on the remaining 19 samples (Table 1).

CircRNAs were predicted from these libraries using three distinct pipelines, CIRCexplorer2 [37], CIRI2 [56] and Hyb [45]. CIRCexplorer2 and CIRI2 are two of the most widely utilized pipelines for circRNA identification and found approximately 82,000 and 180,000 distinct circRNAs across these tissues, respectively (Figure 1b). Over 72,000 circRNAs (88% of CIRCexplorer2 circRNAs and 40% of CIRI2 circRNAs) were found in common between these pipelines, with CIRI2 identifying a larger number of very lowly abundant circRNAs (1 or 2 backsplice junction counts) accounting for the almost 100,000 extra unique circRNAs (Figure S1d). Hyb, a pipeline designed to identify chimeric RNA reads and which was used to identify circRNAs in a previous report [45], was employed here to identify circRNAs genome-wide. In this study, Hyb identified over 73,000 circRNAs; approximately 45% of these were also identified in either CIRCexplorer2 or CIRI2. These trends are consistent with previous reports showing variability across pipelines for predicting circRNAs [44]. Further analyses were focused on the subset of 13,209 high-confidence circRNAs meeting strict thresholds, including normalized expression minimum (CMP > 0.01); a minimum of two unique reads in at least three samples and those commonly identified in all three pipelines.

Interestingly, despite the distinct population of circRNA identified between the three pipelines, the PCA plots for the unfiltered lists are similar and each showed a clear separation between healthy and grade IV (GBM) samples (Figure 1c). However, grade II and grade III tumors did not show a clear separation in any of the analyses. This ability to stratify GBM tumor from normal tissue based on circRNA profile alone suggests that some circRNAs may constitute bona fide biomarkers of GBM. From our high-confidence population of circRNAs averaged within each sample type, we identified circRNA candidates which are altered in expression compared with healthy tissue, for grade II, grade III, and grade IV (GBM) glioma tumors (Figure 1d). There are more circRNAs which are significantly downregulated in GBM compared to healthy tissues. Heat map representation clearly shows that, while some specific circRNA candidates are enriched in healthy tissue or distinct glioma grades, there is biological variability between patients (Figure 1e; Table S2). One example, *circGLIS3*, was also previously shown to be more highly expressed in GBM compared to normal tissue [57]. While beyond the scope of this study, our study supports the finding that these, and other, circRNAs could represent not only diagnostic, but prognostic biomarkers of gliomas and GBM.

### 3.2. Microexons Are Identified in Circrna Sequencing

**Figure 2:**
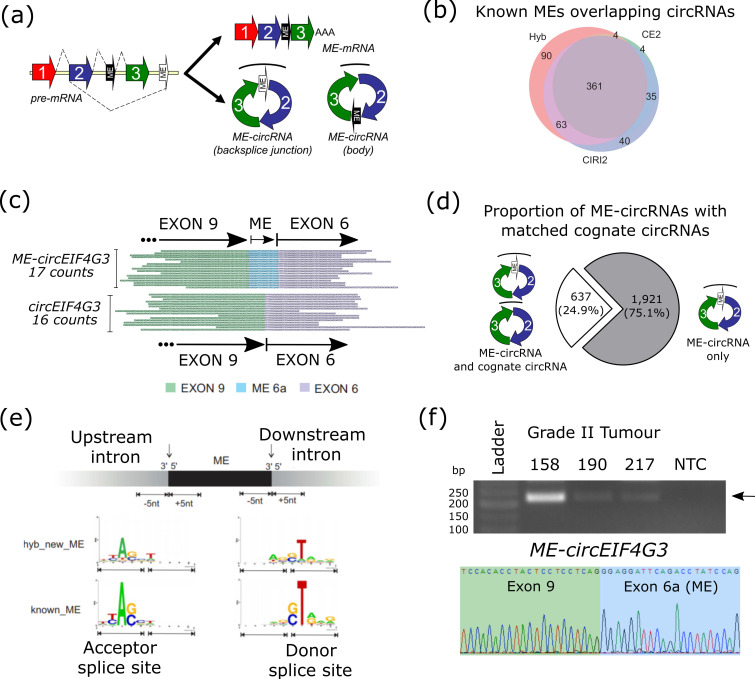
Microexons are included in circRNAs. (**a**) Incorporation of microexons (ME), which are defined here as 3-30 nt sequences into mRNA and circRNA. Note that MEs in circRNAs can fall into the backsplice junction (unfilled exon) or into the body (filled exon) of the circRNA. (**b**) circRNAs overlapping known microexon sequences for three pipelines. (**c**) Trimmed reads mapped to *EIF4G3* which cover the circRNA backsplice junction across exon 9 and exon 6 (*circEIF4G3, 16 counts*) and the same junction with a 21nt ME (*ME-circEIF4G3*, 17 counts) from healthy brain tissue from patient #170. (**d**) *De novo* identification of ME-circRNAs using Hyb. Pie chart showing proportion of ME-circRNAs with matched cognate circRNA (637/2558; 24.9%) and orphan ME-circRNAs (1921/2558; 75.1%). (**e**) Sequence motif enrichment using WebLogo (ver2.8.2) around splice sites of predicted (upper) and known (lower) MEs in ME-circRNAs. The lines under each graph indicates the 5 nt of the intron side and the exon side of the ME splice site with the upstream acceptor splice site on the left and the downstream donor splice site on the right. (**f**) Upper, Agarose gel showing RT-PCR amplification of *ME-circEIF4G3* in RNA from tumor samples #158, #190 and #217 and absence in non-template control (NTC). Lower, Sanger sequencing of PCR product across backsplice junction between the ME (blue shading, exon 6a) and exon 9 (green shading).

While yet to be reported for circRNAs, incorporation of a microexon (ME)—defined herein as an exon comprising 3–30 nt in length—into circRNAs may occur within the body of the circRNA or at the backsplice junction (Figure 2a). Therefore, to see if circRNAs could incorporate MEs, we utilized a reference database of 1239 known MEs localized to specific genomic coordinates within mRNAs from Irimia et al. [15] and searched for overlap with circRNAs from our glioma RNA-seq data. To maximize the accuracy of ME-circRNA prediction, we only included those known MEs whose expression was found in our circRNA-seq data with reads crossing the ME and both flanking exons from the same gene. An example of this is the known ME in *SUPT5H* which is incorporated into reads spanning canonical exons 4-5 (Figure S1a).

After filtering for expression of the ME, we identified 696 of these MEs (or 56%) overlapping our high-confidence circRNAs with, on average, 8.5% of these located at either the 5′ or 3′ exon comprising the circRNA backsplice junction and the remainder within the body of the circRNA (Table S3). As depletion of linear RNA is accomplished by pre-digestion of the RNA with RNase R prior to library preparation (Figure S1b), this suggests that these MEs are not derived from mRNA sequences, but most likely exist within the enriched circRNAs themselves. In support of this, the gold-standard ME in the *REST* mRNA could not be detected following RNase R digestion in any of the healthy or tumor samples, or by RT-PCR (Figure S1b), while *circLRBA* was detectable. Surprisingly, despite ME-circRNAs not being reported to date, all three pipelines identified 361 common ME-circRNAs, covering 29% of known MEs from Irimia et al. [15]. The circRNAs predicted from the Hyb pipeline overlapped with the greatest number of known MEs (518), followed by CIRI (499) and CIRCexplorer2 (404) (Figure 2b).

This is the first report, to our knowledge, of ME-cirRNAs, with CIRCexplorer2, CIRI2, and Hyb identifying previously annotated MEs in the reference genome, rather than new ME-circRNAs. As this indicates there is greater complexity within the cellular repertoire of circRNAs than previously appreciated, we expanded our search to identify de novo MEs in this circRNA-seq data.

### 3.3. Using Hyb Identifies Novel MEs and ME-circRNAs

The Hyb pipeline was originally designed to identify de novo RNA chimeras in an unbiased manner from RNA-seq [45], and has been used to verify 190 of the 239 circRNAs from Memczak et al. [58]. Therefore, we employed Hyb to identify MEs at the backsplice junction of circRNAs. In accordance with previous characterizations, MEs were specified to comprise sequences of 3–30 nt in length between exons which form the circRNA backsplice junction. Across these libraries, Hyb predicted a total of 2,558 ME-circRNAs with the ME at the backsplice junction (Table S4), which is approximately 3.5% of all circRNAs identified using the Hyb pipeline. In a similar result, of the 518 known MEs from Irimia et al. [15] shown to overlap circRNAs using Hyb (Figure 2b), 30 of these were shown to exist at the backsplice junction (Table S3), or 5.8% of known ME-circRNAs.

As the first report of MEs in circRNAs, we performed two in silico analyses to assess the predictive accuracy of the Hyb pipeline. Taking all reads supporting ME-circRNAs using the Hyb pipeline across the 19 brain tissue samples (11,888 reads), we isolated the ME sequence. These were then either (1) randomly shuffled into the backsplice junction of one of the circRNAs (without rearrangement of the ME sequence) or (2) the ME sequences (>6 nt in length) was scrambled and reinserted into the same circRNA. These tests were to mimic an artefactual or random inclusion of a sequence which could arise by chance or library preparation artefact and give rise to false positive ME-circRNAs. We then aligned these shuffled or scrambled ME-circRNAs with the original ME-circRNA reads from the Hyb output. If these shuffled or scrambled ME-circRNA reads were still detected at high levels, then our Hyb pipeline could not be considered highly accurate due to a high false positive rate. On the contrary, only 172 shuffled ME-circRNA reads (1.44%) and 38 scrambled ME-circRNA reads (0.3%) could be found by chance in our ME-circRNA database. This suggests a predictive accuracy exceeding 98.5%, providing confidence in the Hyb pipeline for identifying *bona fide* ME-circRNAs.

One example of a novel ME-circRNA is evident from the trimmed reads from the *EIF4G3* gene (Figure 2c). For this candidate, both the canonical circRNA which has a backsplice junction comprising exon 9 and exon 6 (*circEIF4G3*) and the equivalent ME-circRNA (*ME-circEIF4G3*) incorporating a 21nt ME (called exon 6a) could be detected with 16 and 17 reads, respectively, in the healthy brain tissue of GBM patient #170 and 3 and 0 reads in the matched GBM tumor. This provides evidence that Hyb is the only pipeline currently with the capacity to capture this non-trivial panel of potentially functional ME-circRNA candidates.

For the de novo ME-circRNAs identified by Hyb, we wanted to see how many cognate circRNAs were present which shared the same backsplice junction as a ME-circRNA, but lacking the ME. We found 629 examples (24.9% of ME-circRNAs) where both the circRNA and the ME-circRNA were present (Figure 2d). In addition to *ME-circEIF4G3* (Figure 2c), another example of this was *circRIMS1* which had a canonical circRNA comprising exons 27-29 (chr6: 73016960-73043538) and *ME-circRIMS1* which also incorporates a 30nt ME at the backsplice junction between exons27-29. However, for the remaining ~75% of ME-circRNAs, these did not have a cognate circRNA, suggesting a large proportion of mutually-exclusive circRNA splicing events with respect to MEs.

We sought to identify the most likely genomic coordinates for the novel ME sequences predicted by Hyb in the 2558 ME-circRNAs. Due to small ME sequences mapping at multiple loci, we focused only on MEs 6 nt or longer and wanted to establish whether canonical intron splicing motifs flanked these MEs. For these MEs, the strongest hit was identified by prioritizing following three criteria: matching sequence must be on the same strand as the circRNA, within the same gene and in closest proximity to the 5′ or 3′ exon comprising the canonical circRNA backsplice junction. Once this list of putative coordinates was generated, we performed sequence enrichment motif analysis using WebLogo (ver2.8.2) to assess whether the canonical GT-AG rule for spliced introns is abided by for MEs [59]. By looking at the 5nt either side of both the start and end of the ME, we identified that the dinucleotides at the first two intron positions (donor splice site) and last two intron positions (acceptor splice site) flanking the putative ME were enriched for GT and AG, respectively, providing confidence in our predicted genomic coordinates for these spliced MEs (Figure 2e).

Validation of one putative ME-circRNA identified by the Hyb pipeline—*ME-circEIF4G3* comprising exons 6a-9, with the ME at the 5′ end of the backsplice junction—was undertaken by RT-PCR. RNase R-treated RNA from grade II tumors underwent reverse transcription and RT-PCR using an oligonucleotide anchored in the 21 nt ME itself (exon 6) and another divergent oligonucleotide in exon 10. The 218 bp PCR product corresponding to the ME-circRNA was detected in the tumors and at the same relative abundance of that ME from within each RNA-seq dataset (#158 had 307 ME counts, #190 had 114 and #217 had 130 ME counts), with Sanger sequencing confirming the ME comprises the backsplice junction (Figure 2f). As further evidence for the existence of ME-circRNAs identified by Hyb, we also performed RT-PCR and Sanger sequencing for *ME-circDPY19L1* (Figure S2). Using oligonucleotides anchored in exons 6 and 3 flanking the ME, *ME-circDPY19L1*, unlike *ME-circEIF4G3*, did not exhibit a matched cognate circRNA as there was a single amplicon of the anticipated size when amplified from two different tumor samples.

### 3.4. Decrease in ME-circRNAs in GBM Is Correlated with SRRM4 Expression

Comparing matched normal and GBM datasets we, like others, observed a decrease in the total number of circRNA backsplice junction counts across all three pipelines. Specifically, we identified 32,617 circRNAs in healthy tissues, while only 28,842 circRNAs were detected in GBM tissues (11% decrease). This was similar when looking at the high-confidence subset of 13,209 circRNAs with 11,773 found in healthy tissue and 10,621 in grade IV tumors (9.8% decrease; Figure S3). When focusing on MEs alone and ME-circRNAs identified by Hyb, there was a 22% and 37% decrease between the healthy and grade IV tumors, respectively. Therefore, we sought to identify which protein factors known to regulate ME inclusion in the brain could be correlated with this greater decrease in ME-circRNAs.

**Figure 3:**
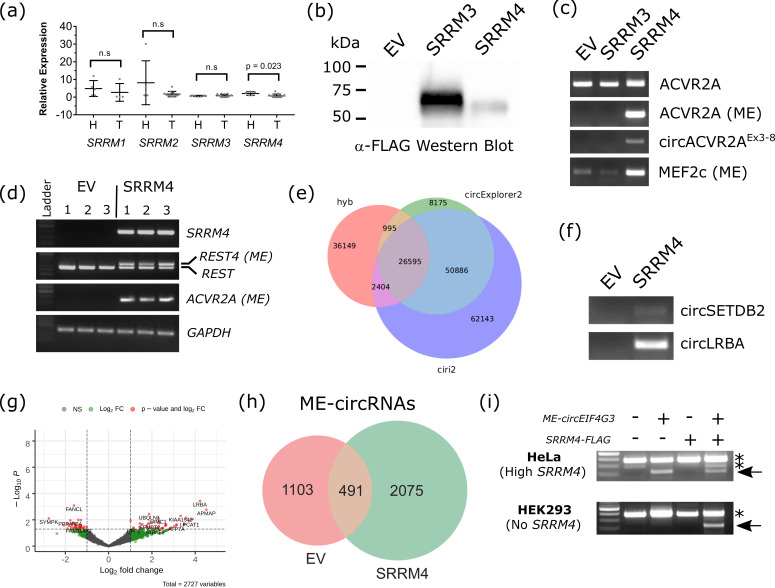
Serine/arginine repetitive matrix 4 (SRRM4) is a *bona fide* circRNA and ME-circRNA biogenesis factor. (**a**) qRT-PCR comparing relative expression of SRRM transcripts between healthy brain tissue (H) and GBM tumor tissue (T). Normalized to GAPDH, n = 4–5 tissue samples. One-way ANOVA, Mann–Whitney test was performed to assess significance. n.s.: no significant difference (*p* > 0.05) (**b**) Western blot using FLAG antibody on cell protein lysates from HEK293 cells transiently transfected with pcDNA3.1 (EV), pcDNA3.1::SRRM3-FLAG (SRRM3) or pcDNA3.1::SRRM4-FLAG (SRRM4). Bands of expected size for SRRM3 (70 kDa) and SRRM4 (65 kDa). (**c**) RT-PCR for *ACVR2A* linear (ACVR2A), and SRRM4-specific *ACVR2A* RNA with ME, *circACVR2A* (comprising exons 3-8) and increase in *MEF2C* linear RNA with ME. (**d**) RT-PCR on 3 biological replicates of HEK293 cells with EV, or SRRM4 overexpression for *SRRM4*, *REST* (2 bands, with upper band representing the inclusion of ME in *REST4*), *ACVR2A (ME)* and *GAPDH* used as loading control. (**e**) Scaled Venn diagram showing total number of circRNAs identified across the three replicate EV and *SRRM4* overexpression circRNA-seq libraries using three different circRNA identification pipelines, CIRCexplorer2, CIRI and Hyb. (**f**) RT-PCR for two novel SRRM4-driven circRNAs showing absence in HEK293 EV cells. (**g**) Volcano plot comparing circRNA expression between EV and *SRRM4* overexpression. (**h**) Scaled Venn diagram showing ME-circRNAs, with the ME present at the backsplice junction, identified using Hyb. (**i**) RT-PCR showing the dependence on SRRM4 for expression of *ME-circEIF4G3* from transgenic circRNA overexpression construct. Top, HeLa cells (with high levels of SRRM4 expression) could produce *ME-circEIF4G3* with and without SRRM4 overexpression 24 h post-transfection. Bottom, HEK293 cells (lacking SRRM4 expression) could only produce the circRNA if SRRM4 was co-transfected. * are non-specific RT-PCR products.

Probing the GEPIA interactive web server, which incorporates over 18,000 matched tumor and normal samples from the TCGA and GTEx projects [60], we identified *SRRM4* was significantly reduced in both GBM tumors and low-grade gliomas compared with healthy tissues (Figure S4a). We also checked the expression of *U2AF2* and *RBFOX1,* two other known factors implicated in neuronal ME splicing [10], by semi-quantitative PCR between the three tumor grades. This showed no clear visible alteration in expression across the grades for *RBFOX1* or *U2AF2*, but *SRRM4* exhibited a noticeable decrease (Figure S4b). Therefore, we performed qRT-PCR on the five healthy and five GBM tissues which we previously sequenced (Table 1) to quantify the expression levels of the known neuronal splicing factor *SRRM4* and its family members *SRRM3* (neuronal-specific) and the ubiquitously-expressed *SRRM1* and *SRRM2*. Comparing expression by qRT-PCR, SRRM4 was the only family member to be significantly decreased (One-way ANOVA, Mann–Whitney test, *p* = 0.023) in expression in GBM compared with the normal brain tissue (Figure 3a). This lower expression of *SRRM4* in the core tumor compared with healthy tissue correlates well with the expression profiling from the publicly available IVYGap database.

### 3.5. SRRM4 Is a Bona Fide circRNA and ME-circRNA Biogenesis Factor

To investigate whether SRRM4 could regulate ME inclusion in circular RNAs, we overexpressed SRRM4 in HEK293 cells as per Torres-Mendez et al. [61] and Nakano et al. [62]. These cells were chosen as they lack *SRRM4* expression, allowing us to delineate the consequence of this gene on circular RNA identity. A C-terminal FLAG-tagged construct was made for *SRRM4* and *SRRM3* (as a control) and transfected into HEK293 cells. This produced a protein of the anticipated size for each construct by western blotting with anti-FLAG antisera (Figure 3b). RT-PCR was performed on matched RNA samples to detect *ACVR2A* mRNA, *ME-ACVR2A* and *circACVR2A*. It was found that while there was not a significant difference in baseline expression of *ACVR2A* mRNA between EV, SRRM3 and SRRM4 overexpressing HEK293 cells, we noted a novel ME-containing amplicon using oligonucleotides that flank the ME (Figure 3c, upper panel) producing the wild-type product (196 bp) in all lines, but a larger, ME-containing band (220 bp) uniquely in the SRRM4 overexpressor. This was confirmed using an oligonucleotide anchored in the ME itself, with the product only seen in the SRRM4 overexpressor line, as expected (Figure 3c). Furthermore, a unique circRNA from *ACVR2A*, comprising exons 3-8, was found in the SRRM4 overexpressor alone (Figure 3c).

RT-PCR on three biological replicates showed the presence of *SRRM4* only in the overexpression lines, not the EV cell lines (Figure 3d). Furthermore, the inclusion of the microexon in the *REST* transcript driven by SRRM4 was investigated by RT-PCR as per Nakano et al. [63]. These primers flank exon 4 of *REST* and produce a 225 bp product from the active *REST* mRNA and a 309 bp product from the *REST4* isoform. In the presence of the empty vector, only the shorter amplicon was detected, while all three biological replicates of SRRM4-FLAG overexpression saw the *REST4* amplicon also detected.

RNA was harvested from HEK293 cells, 48hrs post-transfection with either pcDNA3.1 (empty vector, EV) or pcDNA3.1::SRRM4. CircRNA-seq was performed as for GBM cells on three biological replicates of EV and SRRM4 overexpression and the resultant libraries sequenced on the Illumina NextSeq550. The distribution profile of circRNAs predicted with the three pipelines CIRCexplorer2, CIRI2 and Hyb (Figure 3e) were similar in distribution, but fewer in total numbers, compared with the glioma circRNA-seq (Figure 2b). CircRNAs predicted by Hyb, showed a 10% increase in the count of unique circRNAs identified from 43,380 circRNAs in EV to 47,869 circRNAs with SRRM4 overexpression. Two novel, SRRM4-driven circRNAs—*circLRBA* and *circSETDB2*—were validated by RT-PCR as being absent in HEK293 cells and present in the SRRM4 overexpressing cells (Figure 3f). Furthermore, an enhanced Volcano Plot of this data highlights more circRNAs are quantitatively increased in expression along with SRRM4 in HEK293 cells, rather than decreased or lost (Figure 3g). However, a more significant increase was found in ME-circRNAs (61% increase) with SRRM4 overexpression, including over 2000 unique ME-circRNAs at the backsplice junction (Figure 3h; Table S5). This agrees with SRRM4 being a master regulator of ME inclusion in mRNAs and that it recapitulates these effects with ME-circRNAs. One example of this was a microexon in the *ACVR2A* gene which could be amplified by RT-PCR uniquely in all three SRRM4 overexpression replicates and not in the empty vector (Figure 3c,d). While *MEF2C* mRNA containing a ME was present in EV and SRRM3 lines, it visibly increased in abundance with SRRM4 expression, suggesting SRRM4 can boost ME inclusion when there is a basal level of expression (Figure 3c).

While this evidence shows a number of circRNAs and ME-circRNAs can be regulated by SRRM4 expression, we wanted to validate the dependency of a ME-circRNA on SRRM4 expression. To address this, we focused on a ME-circRNA from our GBM data (Figure 2e), *ME-circEIF4G3*, but notably absent from the HEK293 data. We constructed a circRNA overexpression vector to express *ME-circEIF4G3*, which was transiently transfected into HeLa cells, a cell line known for possessing one of the highest expression levels of *SRRM4* expression from the Human Proteome Atlas [64] (Figure S5a), but did not produce this ME-circRNA de novo (Figure 3i). This is not unexpected, given the cell-type specific nature of circRNAs [65]. An RT-PCR product specific for this circRNA was detected (and confirmed by Sanger sequencing) only upon expression of the *ME-circEIF4G3* transgenic construct (Figure 3i). Co-transfection of the circRNA construct with pcDNA3.1::*SRRM4* in this line did not significantly increase the level of this ME-circRNA. This experiment was repeated in HEK293 cells, which we show lacks expression of SRRM4 (Figure 3c, Figure S5a). Remarkably, despite the use of perfectly reverse complementary introns of 800 bp in length in the overexpression construct, *ME-circEIF4G3* was only produced upon co-transfection with pcDNA3.1::*SRRM4* in HEK293 cells (Figure 3i). This provides strong evidence for the role of SRRM4 as a bona fide circRNA and ME-circRNA biogenesis factor, suggesting it is necessary in both HeLa and HEK293 cells, and sufficient in HEK293 cells for biogenesis of *ME-circEIF4G3*.

## 4. Discussion

To our knowledge, this is the first report of microexons (MEs) detected in circular RNAs. We identified thousands of circRNAs containing MEs, which we deem ME-circRNAs, which were previously unidentified, or unappreciated. These should now be recognized as a non-trivial sub-family of non-coding RNAs and be a focus of new research. In the most comprehensive study to date on diverse circular RNA composition, Zhang et al. [37] identified novel exons being incorporated into circRNAs but did not report MEs as a non-canonical component.

MEs are known to play functional roles in development, with emerging evidence of their association with diseases including autoimmune conditions and cancer [14]. In particular, SRRM4, implicated here as a bona fide circRNA and ME-circRNA biogenesis factor, was shown to regulate a splicing program critical in the trans-differentiation and progression of neuroendocrine prostate cancer [38,39]. MEs within the *EIF4G1* and *EIF4G3* transcripts were shown to be misregulated in autism patients and control synaptic translation and cognition contributing to pathogenesis of this condition [66]. Despite the focus of these reports being on the alternative splicing of the coding transcriptome, our study opens the possibility that ME-circRNAs, like other circRNAs, could play a pathogenic role in cancer. With respect to glioma, we identified a significant correlation (*p* < 0.0001) between low *SRRM4* expression and poor survival in GBM and low-grade glioma across the Chinese Glioma Genome Atlas (CGGA), The Cancer Genome Atlas (TCGA) and Repository of Molecular Brain Neoplasia Data (Rembrandt) datasets (Figure S5b–d). We show here that *ME-circEIF4G3* is controlled by SRRM4 and, given the down-regulation of SRRM4 in gliomas and association with poor survival outcomes, this may warrant further investigation of the role of this and other ME-circRNAs in gliomagenesis.

The expression of SRRM4 in HEK293 cells, which do not express this neural-specific transcript, was a deliberately controlled experiment and with that we found thousands of circRNAs and ME-circRNAs were altered in expression. Interestingly, many of these were intermediate-lowly expressed circRNAs with the four most abundant circRNAs unchanged (*circSPECC1* (Figure S1a), *circMAN1A2*, *circSMARCA5*, and *circHIPK3*). This is also the first report showing a dependence of a transgenic circRNA overexpression construct encoding *circME-EIF4G3*, requiring co-expression of SRRM4 (or in a cell line, like HeLa cells, with high baseline expression of *SRRM4*) to generate the circRNA product. This may be a consequence of the ME perturbing the secondary structure of the RNA, despite the presence of reverse complementary introns which are normally sufficient to drive circRNA expression. Further research will need to be undertaken on the newly identified ME-circRNAs including their unique regulation, impact on RNA secondary structure and function.

## Figures and Tables

**Table 1:** Sample ID, tumor type and grade, RNA sequencing read and alignment statistics. Tumor sample ID #267 (colored in red) was excluded from RNA seq analysis due to low percentage mapped reads.

Sample ID	Tumor Type	Grade	# Input Reads	# Uniquely Mapped Reads
158	Astrocytoma	II	26,343,021	89.1%
190	Oligodendroglioma	II	27,302,075	88.8%
217	Oligodendroglioma	II	25,241,413	84.9%
207	Diffuse glioma	II	23,492,240	88.4%
267	Oligodendroglioma	II	15,437,385	39.1%
150	Anaplastic astrocytoma	III	21,617,416	86.6%
154	Anaplastic oligodendroglioma	III	29,164,440	90.8%
172	Anaplastic oligodendroglioma	III	42,012,355	66.5%
213	Anaplastic astrocytoma	III	25,758,766	87.6%
214	Anaplastic astrocytoma	III	28,412,184	85.7%
169	Glioblastoma	IV	28,268,617	87.6%
170	Glioblastoma	IV	27,580,126	82.7%
203	Glioblastoma	IV	27,106,465	88.4%
61	Glioblastoma	IV	29,197,512	83.0%
160	Glioblastoma	IV	23,140,292	86.4%
179	Control		22,852,102	80.3%
61_MC_NOR	Matched Control		29,806,403	87.3%
158_MC_NOR	Matched Control		28,019,827	88.2%
170_MC_NOR	Matched Control		26,038,920	89.1%
267_MC_NOR	Matched Control		24,372,321	73.3%

## Supplementary Materials

The following are available online at https://www.mdpi.com/2073-4409/9/11/2488/s1, Figures S1–S5. Table S1: Oligonucleotides used in this study. Table S2: Normalized expression levels of circRNAs represented by heatmap. Table S3: Known ME overlap with circRNAs predicted from three pipelines. Table S4: CircRNAs overlapping de novo MEs found using Hyb pipeline in glioma and healthy brain tissue. Table S5: CircRNAs overlapping de novo MEs found using Hyb pipeline in HEK293 cells with SRRM4 overexpression.
