# Supplementary material for: SRRM4 Expands the Repertoire of Circular RNAs by Regulating Microexon Inclusion
Source: Cells. 2020 Nov 16;9(11):2488. doi: 10.3390/cells9112488 (PMC7697094; doi:10.3390/cells9112488)
Supplement: Supplementary file 1 [file cells-09-02488-s001.zip › cells-973971-supplementary/cell-973971 Supplementary Figure.pdf]

cells

MDPI

Supplementary Figures

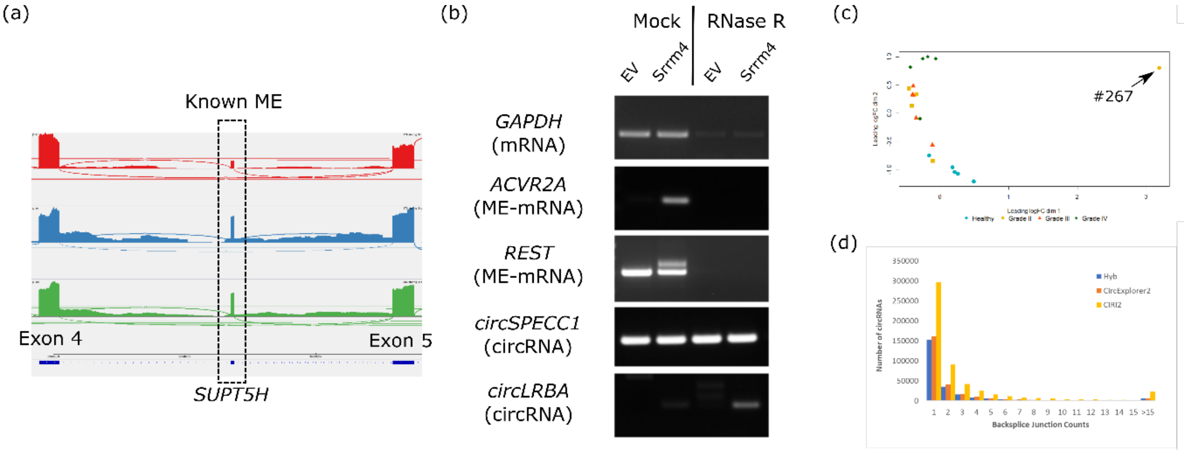

Figure S1. (a) Sashimi plot showing reads aligning to the known ME in SUPT5H and flanking exons 4 and 5 for three samples 179 (healthy, red); 170 (grade IV, blue) and 203 (grade II, green). ME highlighted by dashed box. **(b)** Validation of RNase R digestion. RNA from HEK293 cells with either pcDNA3.1 (empty vector; EV) or pcDNA3.1::Srrm4 (Srrm4) was mock digested or digested with RNase R. RT-PCR was performed for mRNA and circRNA targets with and without microexons (ME). **(c)** Principal component analysis (PCA) plot for circRNAs identified using CIRCexplorer2 for all 20 samples that underwent RNA-seq in thus study. Highlighting outlier sample #267, a grade II glioma which was excluded from further analyses. **(d)** Graph showing counts of circRNAs with various backsplice junction counts identified with three circRNA prediction pipelines Circexplorer2 (orange), CIRI2 (yellow) and Hyb (blue).

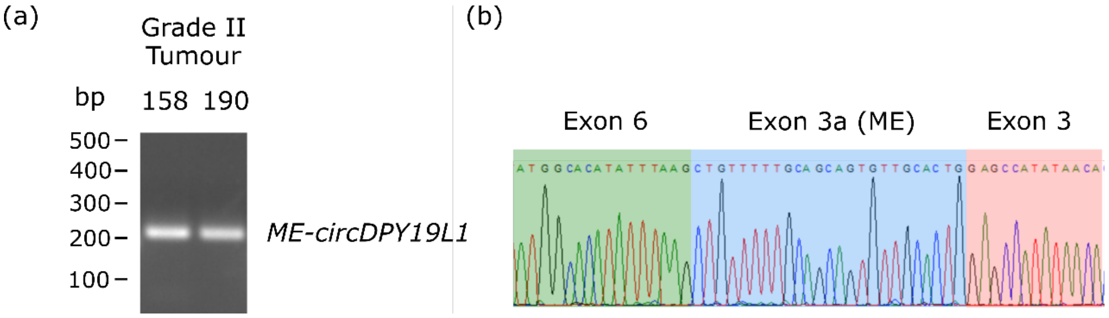

Figure 2. (a) RT-PCR to amplify *ME-circDPY19L1* (hg19 coordinates chr7:35050079-35058208), comprising an microexon of 25nt at the backsplice junction between exon 6 and exon 3 of the *DPY19L1* transcript from grade II tyour samples #158 and #190. **(b)** Sanger sequencing showing ME (blue, labeled as Exon 3a) and flanking exons in ME-circDPY19L1.

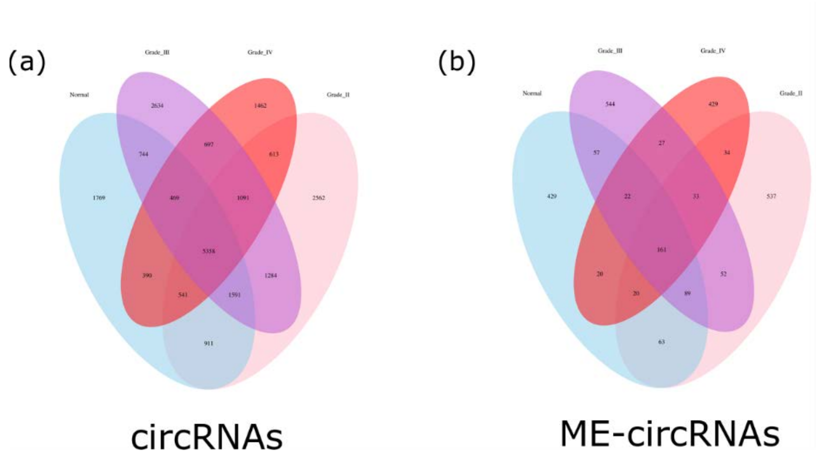

Figure 3. Venn diagrams displaying Hyb output between normal, grade II, grade III and grade IV gliomas for **(a)** circRNAs (high-confidence subset of 13,209 circRNAs) and **(b)** ME-circRNAs.

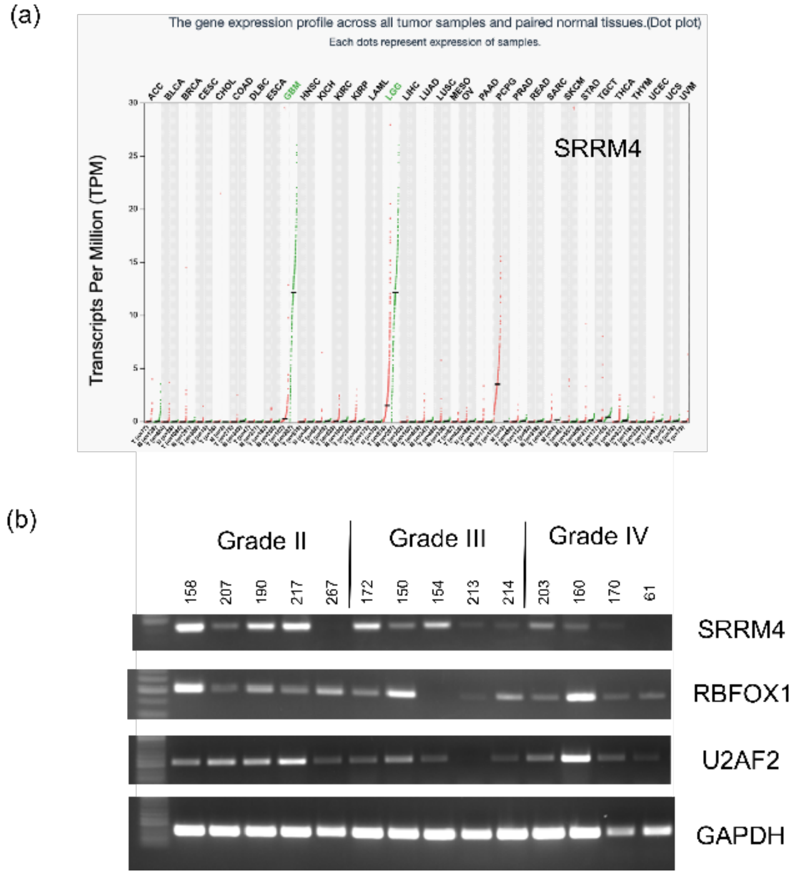

Figure 4. (a) Expression of *SRRM4* in matched tumour and normal samples from the GEPIA directory, showing GBM and low-grade glioma (LGG) are the only sets showing significantly different expression. **(b)** Semi-quantitative RT-PCR for known neuronal splicing factors known to regulate microexon inclusion - *SRRM4, RBFOX1* and *U2AF2* across glioma tumours of varying clinical grading used in this study. *GAPDH* used as loading control.

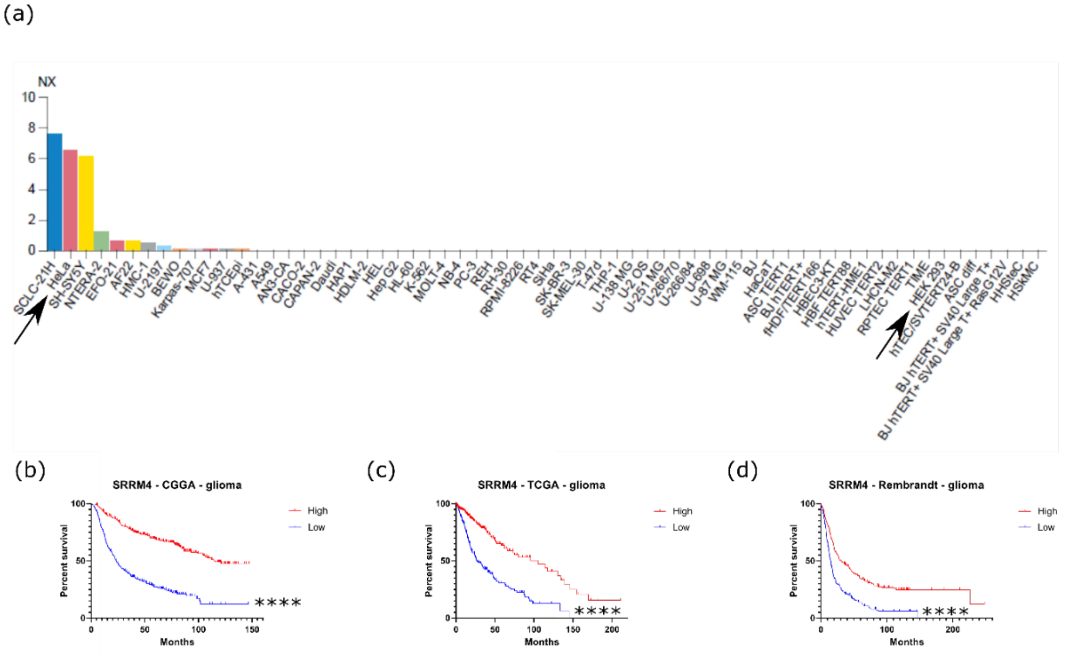

Figure 5. **(a)** Human Proteome Atlas expression profiling of *SRRM4* across human cell lines. Arrows denote HeLa and HEK293 cells as used in this study. Survival plot for high and low expression of *SRRM4* across human gliomas (low-grade glioma and GBM) from the **(b)** Chinese Glioma Genome Atlas (CGGA), **(c)** The Cancer Genome Atlas (TCGA) and **(d)** Repository of Molecular Brain Neoplasia Data (Rembrandt) datasets downloaded from GLioVis. Statistical analyses performed by Log-rank (Mantel Cox) test (****, P-value < 0.0001).
